# Soil Properties Drive Microbial Community Structure in a Large Scale Transect in South Eastern Australia

**DOI:** 10.1038/s41598-018-30005-8

**Published:** 2018-08-06

**Authors:** Pei-Pei Xue, Yolima Carrillo, Vanessa Pino, Budiman Minasny, Alex. B. McBratney

**Affiliations:** 1 School of Life and Environmental Sciences and Sydney Institute of Agriculture, The University of Sydney, Eveleigh, NSW 2015 Australia; 2 Hawkesbury Institute for the Environment, Western Sydney University, Richmond, NSW 2753 Australia

## Abstract

Soil microbial communities directly affect soil functionality through their roles in the cycling of soil nutrients and carbon storage. Microbial communities vary substantially in space and time, between soil types and under different land management. The mechanisms that control the spatial distributions of soil microbes are largely unknown as we have not been able to adequately upscale a detailed analysis of the microbiome in a few grams of soil to that of a catchment, region or continent. Here we reveal that soil microbes along a 1000 km transect have unique spatial structures that are governed mainly by soil properties. The soil microbial community assessed using Phospholipid Fatty Acids showed a strong gradient along the latitude gradient across New South Wales, Australia. We found that soil properties contributed the most to the microbial distribution, while other environmental factors (e.g., temperature, elevation) showed lesser impact. Agricultural activities reduced the variation of the microbial communities, however, its influence was local and much less than the overall influence of soil properties. The ability to predict the soil and environmental factors that control microbial distribution will allow us to predict how future soil and environmental change will affect the spatial distribution of microbes.

## Introduction

Soil microbial communities have an important role in regulating nutrient cycling^1,2^, carbon mineralisation and stabilisation^3,4^. Recent studies have demonstrated that the belowground microbial communities are acting as regional drivers of the aboveground biotic communities such as plant species diversity and productivity^5–7^. Moreover, microbial diversity and composition are widely recognized as key factors in driving ecological functions^8–12^. Thus, it is essential to better understand the causes and controls of soil microbial distribution and composition^13,14^. Various studies at the plot scale have found significant effects of biotic and abiotic factors on belowground microbial composition including land management^15,16^ and its configuration^17^, aboveground biotic diversity and density^18,19^, and microbial successional stage^20^. Recently, there has been an increasing interest in the large-scale biogeographic pattern of microbial distribution. Griffiths *et al*. studied the biogeographic distribution of soil bacteria across the UK^21^. Later on, Terrat *et al*.^22^ explored the geographical bacterial diversity in France. However, the information on microbial patterns at larger scales (e.g., continents, regions) is still limited, especially in Australia.

Previous studies have demonstrated a significant dependency of the biogeographic distribution of soil microbes on environmental filters, such as soil properties, environment conditions (e.g. climate and topography), and even land cover^21–23^. Nonetheless, the reported evidence has not been conclusive in determining the main controlling factors. The spatial regulators of soil biota vary at different spatial scales and in different ecosystems^24^. For instance, Dequiedt, *et al*.^25^ found that the spatial distribution of bacterial communities was more related to soil properties and land cover compared to topography and climate in some regions in France. Other authors reported that the biogeographic variation of soil microbes was more associated with precipitation and soil factors across grasslands on the Mongolian plateau in China^26^. Thus, evidence on the environmental filters is inconclusive, and these different studies that explored different gradients and/or spatial scales resulted in divergent conclusions.

Anthropogenic disturbance via land use and agricultural activities also pose great impacts to the structure of the soil microorganisms. Studies have demonstrated that agricultural practices affect the composition of soil microbiota^27,28^. For example, land use and land cover have been found as the determinant factor affecting soil organic matter content, and thus, regulating the microbial structure accordingly^29,30^. Some studies even suggested that agricultural practices had greater impacts to soil microbial communities than precipitation and elevation gradients^31^ or aboveground vegetation and soil properties^32^. The impact of human activities through land use on the soil microbial composition at large spatial scales are not well understood in their variability across all different agro-ecological environments.

**Figure 1:**
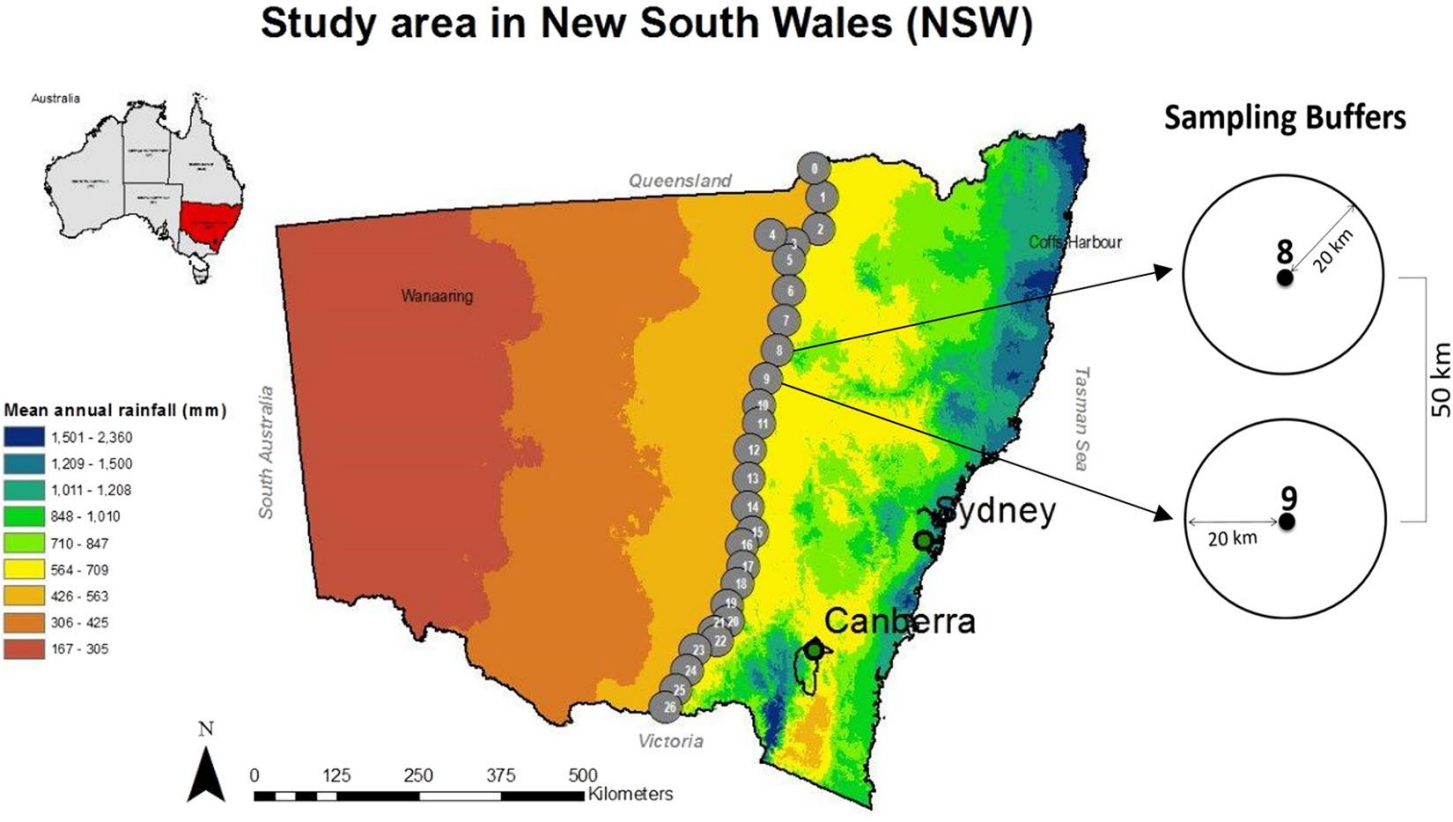
Sample locations along the 550 mm mean annual rainfall isohyet from North to South in NSW. For each location, soils were sampled from paired ecosystems: a disturbed system (i.e., cropping or grazing regions) and an undisturbed system (i.e., natural vegetation, such as forest, grassland or woodlands without human management).

To understand the complex interaction between soil properties, the environment, and agricultural activities, we surveyed the soil microbial community along the 550 mm mean annual rainfall isohyet 1000 km transect in New South Wales (NSW), Australia (Fig. 1). This study aimed to investigate the role of soil properties, climate (soil surface temperature), topography (latitude, slope, elevation), and the above vegetation (Normalized Difference Vegetation Index or NDVI) in determining the structure of the soil microbial communities. The transect was designed to represent a latitudinal agroecological gradient but also to encompass most of the rainfed agricultural area in NSW. A 550 mm isohyet enabled to encompass the Wheat Belt region – the most extensive and economically productive agricultural zone in Australia. Furthermore, through paired site sampling – disturbed (agriculture fields) and undisturbed (natural lands without human management) ecosystems, we assessed how agricultural activities influenced the role of latitude in shaping the belowground microbial communities. Information on the driving factors for the structure of microbial communities can help in understanding the belowground ecosystem and can provide information about the ecosystem response to environmental changes^33^.

## Results

### Soil microbial abundances

**Figure 2:**
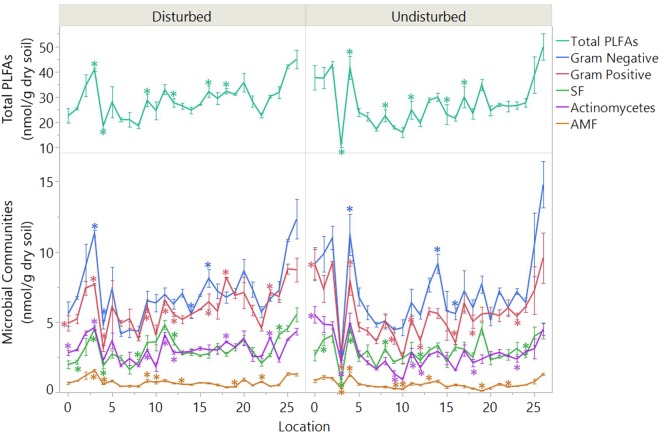
Total PLFAs and biomarkers for each microbial group along the transect. Values are means of concentration (nmol/g dry soil) with error bars representing standard error (n = 3); asterisks (*) denote significant differences between disturbed and undisturbed in the specific site (p ≤ 0.05). PLFAs = Phospholipid Fatty Acids; SF = saprotrophic fungi; AMF = arbuscular mycorrhizal fungi.

The soil microbial composition was assessed with Phospholipid Fatty Acids (PLFAs) analysis. The abundances of the microbial groups were quantified by the abundances of their biomarkers in the form of fatty acid methyl esters (FAMEs). In general, the total PLFAs, an index of the size of the microbial community ranged from 11.1 to 49.8 nmol/g dry soil along the latitude (Fig. 2). There was a great fluctuation from location 3 (Latitude −29.54, *Moree*) to location 6 (Latitude −30.49*, Gwabegar*). The lowest microbial abundance was also detected in these regions, location 3 in the undisturbed system and location 4 in the disturbed system. It was followed by a relatively lower level in the intermediate regions from location 6 to location 22 (*Gwabegar* to *Forest Hill* at latitude −30.49 to −35.18). Total PLFAs then increased noticeably close to the southern border (location 23 to location 26). Similar trends were found in the absolute abundances of major functional groups: gram-negative bacteria, gram-positive bacteria, saprotrophic fungi (SF), actinomycetes and less markedly in the arbuscular mycorrhizal fungi (Mycorrhiza/AMF) (Fig. 2).

Through the multiple comparison (Tukey HSD) analysis within all the sites along the gradient, total PLFAs showed more significant variations in the undisturbed ecosystem (Table S2 in the supplement). In most of the microbial groups, except for actinomycetes, more similarities of the microbial distribution (absolute abundances) were found from location 6 to 22, the middle regions with intensive agriculture practice. When the land was disturbed, the differences of the microbial absolute abundances between the middle region and the northern border (location 0 to 2) were generally decreased compared to undisturbed systems.

Through the paired comparison of each site, significant increases of the microbial absolute abundances after disturbances were generally observed in the middle regions (Fig. 2), like gram-positive bacteria in location 9, 11, 12, 16,18, actinomycetes in site 9, 11, 12, 16, 18, AMF in location 9, 10, 19 and so on. However, the sizes of gram-positive bacteria and actinomycetes (at location 0), as well as SF (at location 2) were lower in the disturbed lands in the northern border (Fig. 2).

### Relative microbial abundance

**Figure 3:**
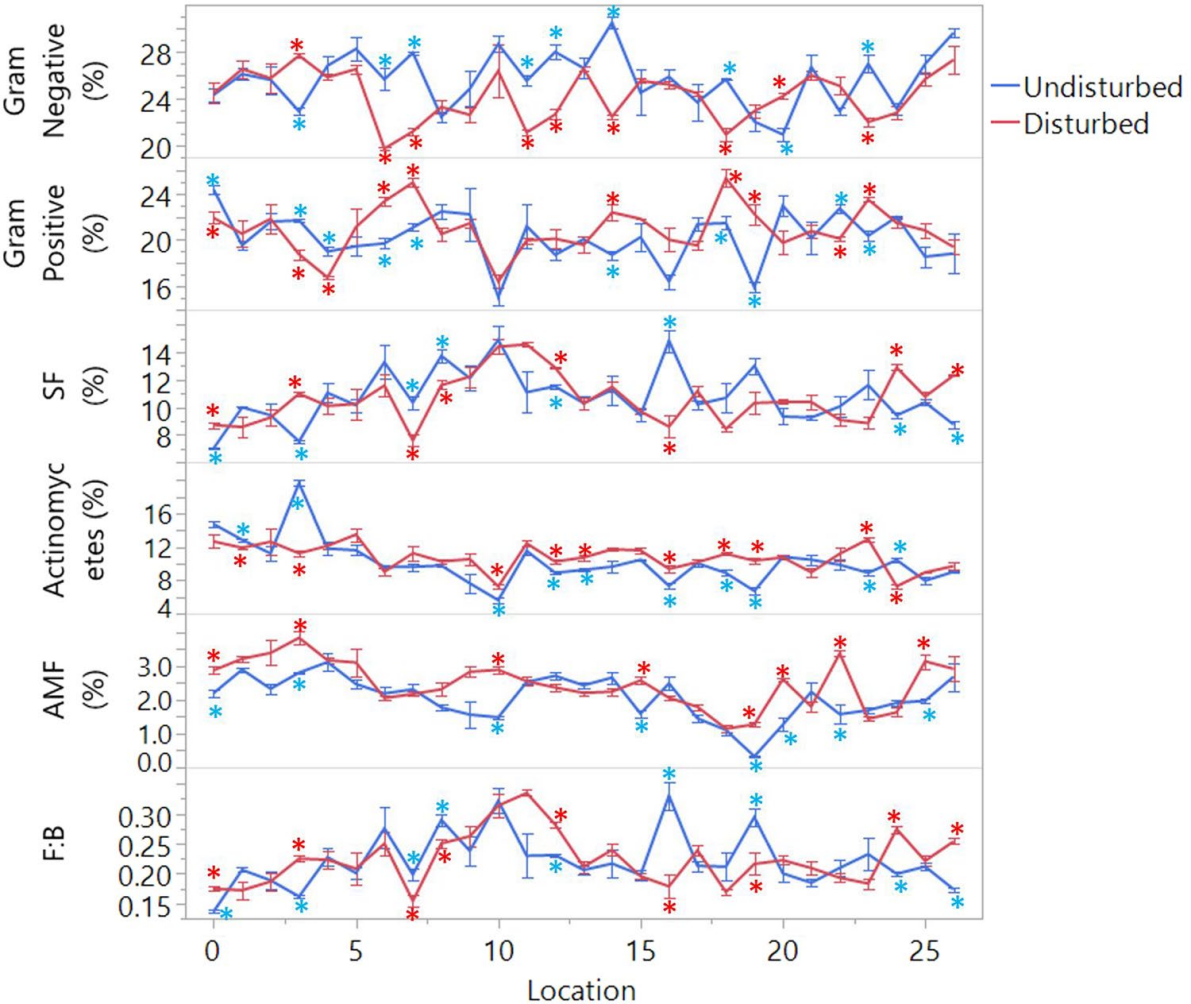
Comparison of the microbial biomarkers in proportion and F:B ratio along the transect in two ecosystems. Error bars represent standard error of the proportion means (n = 3); asterisks (*) denote the significant differences between the disturbed and undisturbed in the specific site (p ≤ 0.05). SF = saprotrophic fungi; AMF = arbuscular mycorrhizal fungi, F: B was calculated by dividing saprotrophic fungal PLFAs by the sum of gram-positive, gram-negative and unclassified bacterial PLFAs.

There was no overall trend observed across latitude for the relative microbial abundances (Fig. 3). Along the transect, gram-negative bacteria accounted for the largest proportion (more than 20%), followed by gram-positive, SF and actinomycetes. AMF showed the least proportion between 0.3% and 3.5%.

Agricultural activities showed some influences on the microbial composition across the latitude, but the influences were not unique. Through the paired comparison, disturbances appeared to cause significant increases in the proportion of gram-positive bacteria, actinomycetes, and AMF in some middle sites. For instance, gram-positive bacteria in location 14, 18, 19 and 23, actinomycetes in 10, 12, 13, 16, 18, 19, AMF in 10, 15, 19, 20, 22 were increased significantly. Significant increases of human disturbances also were present in the proportion of SF and AMF in both the borders, such as SF in site 0, 24, AMF in site 0 and 25. Agricultural impacts on the ratio of fungi: bacteria (F:B) were more region-specific, which was increased in location 0, 3, 12, 24 and 26, but decreased in site 7, 8, 16 and 19.

**Figure 4:**
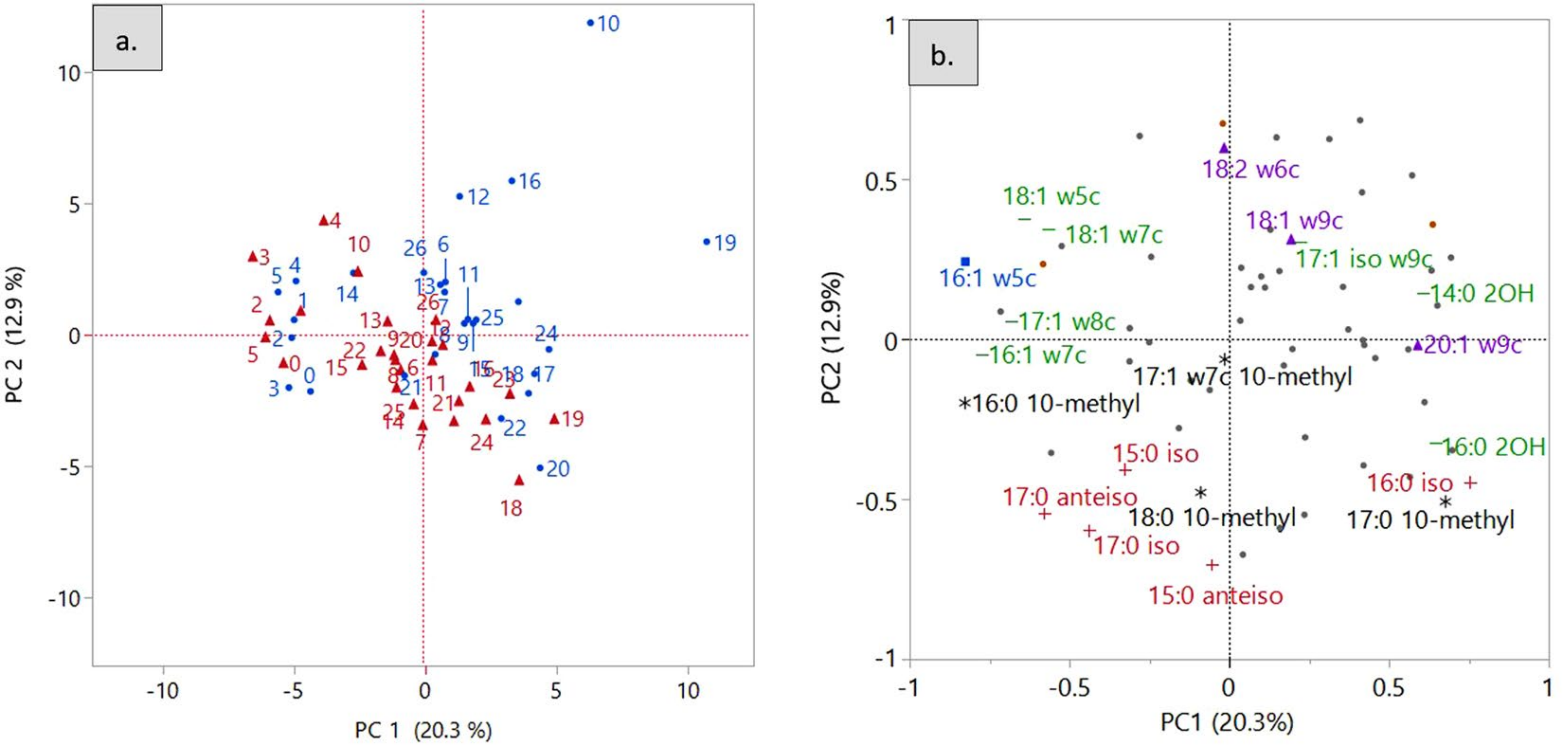
Plots of the first and second principal components (PC1 and PC2) from PCA of molecular weight percentages of whole communities’ PLFA profiles along the transect in the two ecosystems (**a**) and contributions of specific PLFAs (**b**). Red triangle (): disturbed system; Blue dot (): undisturbed system. “  ”: biomarkers for gram-positive bacteria; “” for gram-negative bacteria; “*” for actinomycetes; “” for SF; “” for AMF.

To capture variation in the relative abundance of all the detected PLFAs across both types of ecosystems (65 detected lipids), a principal component analysis (PCA) was conducted. 20.3% and 12.9% of the variations were explained by the first two components respectively (Fig. 4). Overall there was no clear separation of the disturbed and undisturbed soil samples, but some sites, such as undisturbed soils from middle latitude locations 10, 12, 16, 19 were separated in the first quadrant with positive principal component 1 (PC 1) and positive principal component 2 (PC 2) values (Fig. 4a). Accordingly, they had high loadings of SF (18:1ω9c) and gram-negative bacteria biomarkers (17:1 iso ω9c, 14:0 2OH) (Fig. 4b).

**Figure 5:**
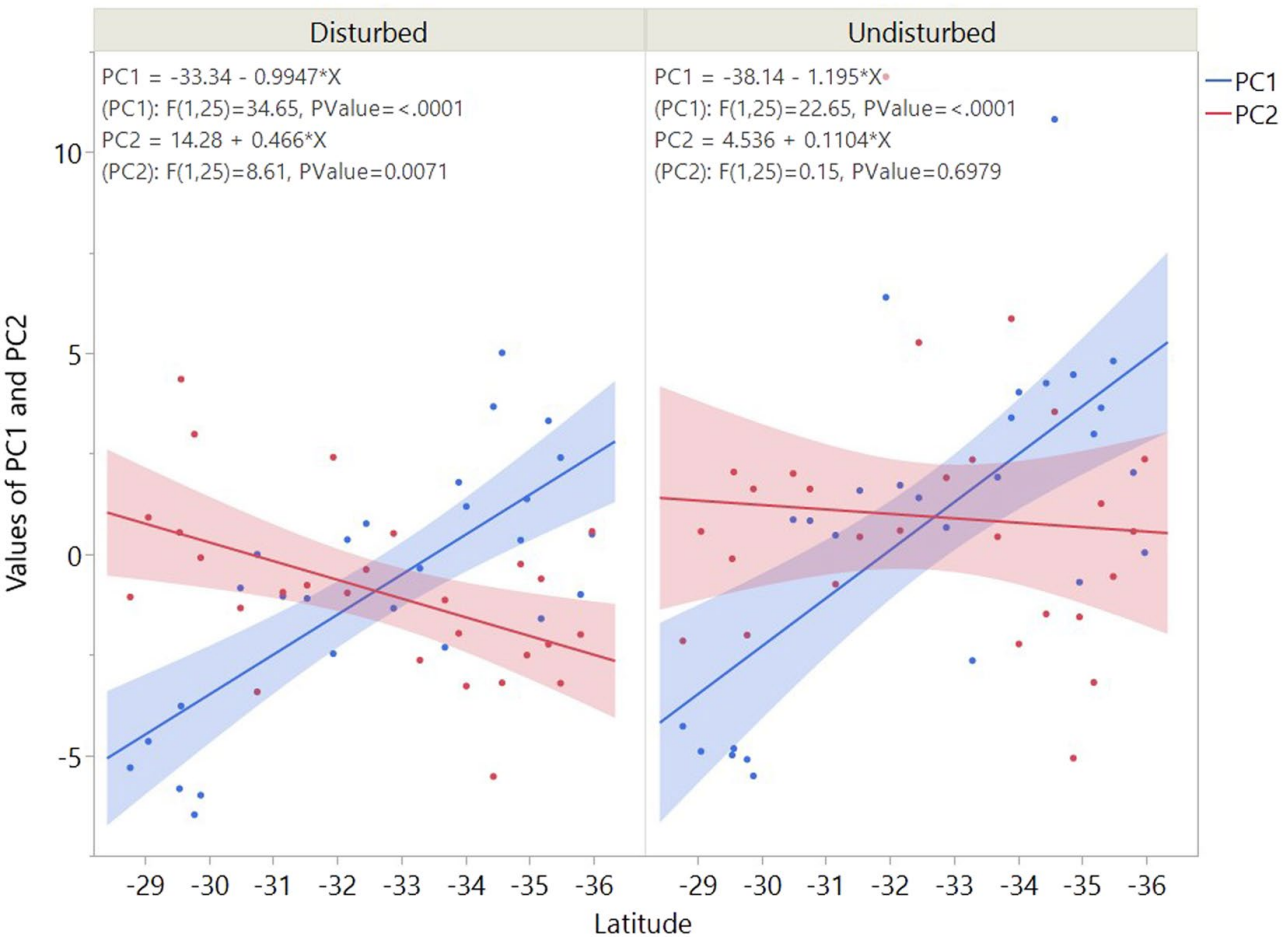
Linear regression between the first two principal components (PC1 and PC2) locations of samples and latitude. P values less than 0.01 were considered to be significantly related. The blue lines represent PC1, and the red lines represent PC2.

Full community PLFAs profiles were influenced by latitude. The principal components were observed to be significantly related to the latitudinal location of soil samples (Fig. 5). Strong positive linear relationships were observed between latitude and PC1 in both ecosystems (Fig. 5). Higher latitude was associated with a greater relative abundance of the biomarkers of SF (18:1 ω9c, 20:1 ω9C), gram-negative bacteria (17:1 iso ω9c, 14:0 2OH, 16:0 2OH), gram-positive (16:0 iso) and actinomycete (17:0 10-methyl) (Fig. 4b). While most of the gram-positive biomarkers, e.g., 15:0 iso, 15:0 anteiso, 17:0 iso, 17:0 anteiso, were less abundant as towards the south, in the higher latitudes. The biomarkers of actinomycetes such as 17:1 ω7c 10-methyl, 16:0 10-methyl were decreasing towards the south. Same trends were detected for 18:1 ω5c, 18:1 ω7c, 17:1 ω8c, 16:1 ω7c the gram-negative biomarkers and 16:1 ω5c the AMF biomarkers (Fig. 4b).

The significant negative influence of latitude on PC2 was observed in the disturbed system but not in the natural ecosystem (Fig. 5). Thus, in the disturbed lands, as latitude decreased, the relative abundance of some gram-negative bacterial markers (i.e. 18:1ω5c, 18:1ω7c, 17:1 iso ω9c, 14:0 2OH, 17:1ω8c), SF (i.e., 18:2ω6c, 18:1ω9c) and AMF (i.e., 16:1ω5c) increased the accumulation of PC2 (Fig. 4b). While biomarkers of gram-positive bacteria (15:0 iso, 17:0 anteiso, 17:0 iso, 16:0 iso, 15:0 anteiso) and actinomycetes (17:1 ω7c 10-methyl, 16:0 10-methyl, 18:0 10-methyl, 17:0 10-methyl) showed opposite trends.

### The Influencing Factors

The Pearson’s linear Correlation Coefficient was calculated between the microbial groups and the possible influencing factors: soil properties and the environmental factors. Soil properties included soil texture (clay and sand contents), pH, nutrients (total C, total N, C:N, soil P, K), Cation Exchange Capacity (ECEC), Electrical Conductivity (EC) and so on. The environmental factors of climate (soil surface temperature), geographical variables (i.e. latitude, slope, and elevation), above vegetation (NDVI) were considered in this investigation. The complete results of the Pearson’s linear Correlation Coefficient were showed in the supplementary documents (Tables S3 and S4). Soil properties, especially the nutrients (e.g. total C, total N, P, ECEC) were found more related with the absolute abundances of the microbial groups (Table S3). While other environmental factors (e.g. latitude, elevation, soil surface temperature and NDVI), as well as soil texture, pH, EC were more correlated with the proportions of the microbial groups (Table S4). Correlations between the microbial richness (absolute abundances) and soil properties were listed in Table 1 below, and significant differences between the two ecosystems were observed.

**Table 1:** Linear correlation coefficients between the microbial absolute abundances (nmol/g dry soil) of each group and the environmental factors.

	Total C	Total N	P (Colwell)	ECEC	pH (CaCl_2_)	Clay Content
Undisturbed Ecosystem						
Total PLFAs	0.36	0.42	***0.50***	***0.50***	0.23	0.32
Gram-Positive	0.20	0.29	0.47	***0.49***	0.29	0.39
Gram-Negative	0.33	0.39	***0.49***	0.48	0.26	0.32
Actinomycetes	0.01	0.09	***0.61***	***0.61***	***0.55***	***0.63***
SF	***0.49***	0.47	0.36	0.32	-0.03	0.09
AMF	-0.03	0.05	***0.51***	0.47	***0.50***	0.44
F:B	0.14	0.00	-0.37	-0.43	-***0.57***	-0.42
Disturbed Ecosystem						
Total PLFAs	***0.58***	***0.60***	0.26	0.13	-0.03	-0.03
Gram-Positive	***0.55***	***0.59***	0.06	0.06	-0.23	-0.04
Gram-Negative	***0.57***	***0.57***	0.36	0.31	0.19	0.09
Actinomycetes	0.35	0.38	0.00	0.37	0.13	0.33
SF	0.48	0.46	0.39	-0.06	-0.05	-0.18
AMF	0.18	0.17	0.34	***0.52***	***0.52***	0.33
F:B	-0.01	-0.06	0.29	-0.36	-0.09	-0.32

^*^PLFAs = Phospholipid Fatty Acids; SF = saprotrophic fungi; AMF = arbuscular mycorrhizal fungi; ECEC = Cation Exchange Capacity.

^*^Significant correlations are highlighted in *Italic bold* (P ≤ 0.01) in accordance with a Pearson’s paired sample association test.

Significant positive influences of soil nutrients were observed in most microbial group in both ecosystems (Table 1). In the undisturbed systems, the microbial groups were more related with soil P, except for gram-positive bacteria and SF groups. ECEC also showed positive relationship with the total PLFAs, gram-positive bacteria, and actinomycetes in the undisturbed soils. However, in the disturbed soils, they were mainly correlated with total C and total N, especially the gram-positive and gram-negative bacteria.

The contributions of various factors, as well as human disturbances, on the community abundance, were identified using a multiple linear regression model. Soil properties, environmental factors, and land use (human disturbances) were considered altogether. The significant influences of the influencing factors were assessed after selection via a stepwise regression approach. The redundant variables were removed manually before the multilinear analysis if the selected variables were highly correlated. Thus, total C was removed for its high correlation with total N (r = 0.97, p < 0.001) in the model of total PLFAs, gram-positive bacteria and gram-negative bacteria. ECEC was removed in the assessment of actinomycetes for its high correlation with clay content (r = 0.79, p < 0.001). Accordingly, the influencing degree of the influencing factors were estimated in Table 2 below.

**Table 2:** Linear Model Parameter estimates between the microbial absolute abundances (nmol/g dry soil) and the influencing factors.

	EC	Total N	Latitude	Clay Content	pH(CaCl_2_)	P(Colwell)	C:N	Sand Content	Elevation	Disturbance
Total PLFAs	-52.76**	15.85**	-2.12***	0.32***		0.14***				
Gram-positive	-9.77**	2.09*	-0.49***	0.07***		0.02**				
Gram-negative	-13.26**	4.81**	-0.55**	0.09***		0.04***				
Actinomycetes	-5.07*		-0.22**	0.05***		0.01**	-0.13**			
SF	-6.46**	2.64***				0.02***				
AMF	-1.61*		-0.07**		0.25**				0.00**	
F:B							0.01***	0.00***		0.01*

Note: 0.01 ≤ p ≤ 0.05 were marked with *; 0.001 ≤ p ≤ 0.1 were marked with **; p ≤ 0.001 were marked with ***. However, p values ≤ 0.01 were considered to be significant.

Generally, the influences of soil EC and total N ranked the highest two factors (Table 2). Significant negative influences of EC were found in most of the microbial groups, except for actinomycetes and AMF. Soil total N explained the significant variation of the absolute abundances of total PLFAs, gram-negative bacteria, and SF. The significant impact of latitude was detected in all the microbial groups except for SF. However, compared with total N and EC, the contribution of latitude to the richness of microbial groups was relatively small. The influence of clay content was next to latitude, but it was positively related with most of the microbial groups. The significant influence of soil pH was only observed in the AMF community, while soil P affected all the microbial groups except for AMF. C:N showed a low negative influence on the richness of actinomycetes, but a slight positive impact on the ratio of F:B. Though sand significantly influenced F:B and elevation showed significant relationship with AMF abundances, the influencing degrees were quite low. However, in this integrated model, the influences of agriculture disturbances were insignificant.

### Comparisons of the soil properties in the two ecosystems

**Figure 6:**
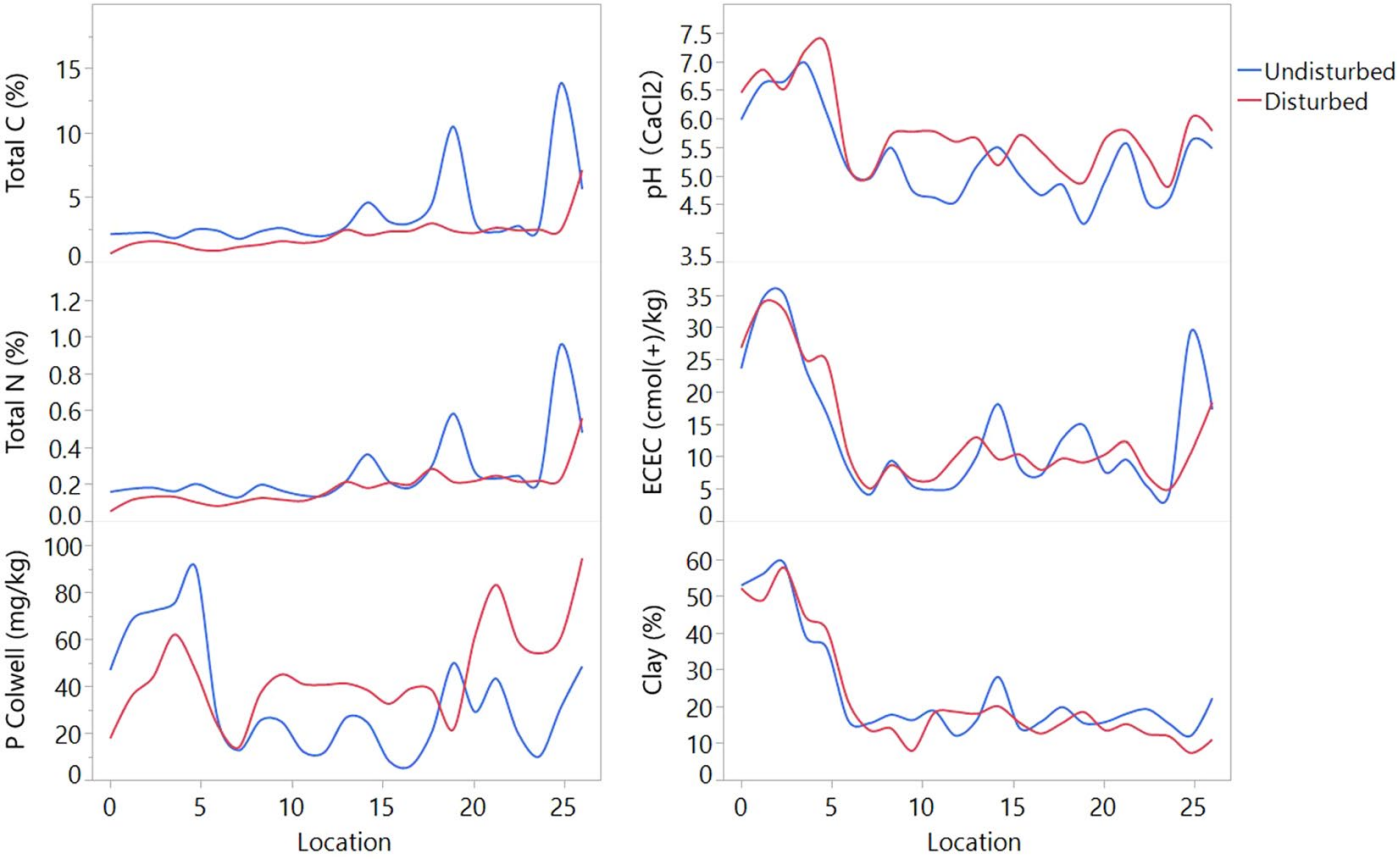
Soil properties’ variation, expressed as smoothed splines (smoothing value = 0.00015), along the 550 mm isohyet transect from the northern border to the southern border of NSW. The blue lines represent the undisturbed ecosystem, and the red lines represent the disturbed ecosystem.

Soil total C and total N behaved in a similar way along the transect and were both lower in the disturbed ecosystems^34^ (Fig. 6). Soil P was higher in most of the disturbed regions but not at all locations. The general trend of soil pH, ECEC and clay content were quite similar in the two ecosystems, with larger values in the northern sites. Soil pH was significantly higher in most of the disturbed sites. The highest pH in the disturbed system was 7.6 at location 4. While in the undisturbed soils, it was 7.5 at location 3. The impact of disturbances on soil ECEC was not consistent along the transect, but the values were less variable in the disturbed soil. Location 0 to 5 are *Vertosols* (Australian Soil Classification System) or *Vertisols* (World Reference Base), which, as expected, had larger clay content^34^.

## Discussion

This study aimed to investigate the role of soil properties and environmental variables in determining the structure of the soil microbial communities across the latitudinal gradient and to assess how agricultural activities influenced the role of soil and environment in shaping the belowground microbial communities.

### The influences of Soil Properties and Environmental Factors

The regional distribution based on the abundance of soil PLFAs markers were found significantly related to latitude across NSW (Fig. 5). This latitudinal distribution can be the result of variations of soil properties and gradients of the environmental factors along the transect as those factors influenced the microbial groups significantly (Table 2). A similar relationship at a similar scale had also been found by other studies^22,24,35^. The main drivers of the soil microbial structure differed amongst microbial groups, but soil properties in terms of soil nutrients, EC, clay content and pH explained most of the variations in this study area (Table 2).

Different soil types proved to be an important factor driving the spatial variation of soil physicochemical properties along this isohyet^36^. The northern sites (location 0 to 5) was dominated by the *Vertisols*, which had a high content of shrink-swell clay, larger pH, and ECEC. Accordingly, higher abundance of the belowground communities was observed in these *Vertisols* (location 0 to 6, latitude -28.77 to 30.49) (Fig. 2). Therefore, the microbial distribution patterns in those area were generally different with other regions, especially in the undisturbed soils (Table S2). This result agreed with the finding of Pino’s study^34^ highlighting the importance of soil types to the soil microbial communities^36^.

The importance of soil nutrients to the soil microbial abundances has been demonstrated in studies that used throughput sequencing techniques^21,23^. In this transect study, the significant correlations between soil nutrients (total N, total C, P, ECEC) and microbial absolute abundances was also observed across the transect (Table 1). Higher levels of soil nutrients were observed in both borders, i.e. higher P, ECEC in the northern regions, and higher C, N, and P levels (Fig. 6). Correspondingly, the size of the microbial communities was higher towards the borders (Fig. 2).

However, other parameters, such as soil pH, EC, latitude, elevation, soil surface temperature, that regulate the belowground living condition were more related with the proportional distribution of the microbial groups (Table S4). Different microbes prefer different living conditions; thus, the variance of the microbial living condition leads to the different vigour of the microbial groups and then influenced the composition of the microbial communities^37^. The F:B ratio was significantly positive with C:N values. This could be explained by the finding that fungi had a competitive advantage over bacteria under low organic conditions^38^.

Soil pH has been widely reported as the overriding factor in determining the soil microbial structure^39^. An extreme pH condition (high or low) can damage the microbial cells and decrease microbial abundances^40^. Certainly, the largest pH conditions in both ecosystems resulted in the lowest microbial abundances along the transect (Figs 2, 6). The variance of the microbes’ pH tolerance regulates the microbial distribution. With the pH ranged between 4.0 to 7.6 in this study, only AMF were sensitive with soil pH in the integrated model. Moreover, the microbial distribution appeared to be an integrated result from all of the environmental and edaphic factors. The impacts of soil pH can be moderated by other factors and resulted in the relatively low correlations with the microbial abundances (Table 2).

An interesting finding was that EC had large influences on the major microbial groups (gram-positive bacteria, gram-negative bacteria, and SF) (Table 2). Soil EC mostly reflects clay content, salt content, and mineralogy, which determine the living condition of the microbes in the soil matrix, like the availability of water, air, substrates and so on. Torsvik and Øvreås^41^ reported that more than 80% of the bacteria were inhabited in the soil micropores, thus, both the gram positive and gram negative bacteria were strongly influenced by soil EC.

Temperature is commonly reported to influence the microbial activity and composition^42^. Correspondingly, significant correlations were found between temperature and the proportion of actinomycetes and AMF. However, the correlations with other microbial communities was insignificant. Temperature effects might be diluted by other stronger factors (e.g., soil nutrients). Moreover, the temperature variation along this transect was not significant enough to promote changes in the community structure.

### The role of land use

Compared with the gradient influences of soil properties, the impact of human disturbance was insignificant (Table 2). However, human disturbances decreased variations of the microbial abundances across the latitude (Table S2) but strengthened the relationship between latitude and the microbial biomarkers (Fig. 5). The influences of land use on the microorganisms could be ascribed to the altering of soil properties via agriculture disturbances.

In the disturbed system, the regional microbial component distributions were found to be more closely associated with soil fertility (total C and N) (Table 1). This could be a signal of the C and N loss by agriculture, as have been investigated by many studies^43,44^. Correspondingly, our data showed that the C and N content under undisturbed system was relatively higher along the transect compared to the disturbed lands (Fig. 6). Despite these, P was found strongly related to the microbial abundances in the undisturbed system but not in the croplands representing disturbed ecosystems (Table 1). Especially for the gram-negative bacteria, actinomycetes, and AMF, the positive relationship with P disappeared in the disturbed ecosystem. This was in accordance, although in terms of abundance in this case, with the study of Delgado-Baquerizo^45^ where the bacterial diversity was reported to be strongly positively related with the availability of P. The low correlation factors in the disturbed soils between P and microbial communities indicated a possible P surplus by human disturbances as P levels were mostly higher in the disturbed soils (Fig. 6). Agricultural activities also smoothed the fluctuation of ECEC variations (Fig. 6), which decreased the influences of ECEC on the communities as a result (Table 1). These variations could be caused by the application of fertilizer. Wenhui, *et al*.^46^ studied a long-term application of the same type fertilizer and confirmed that after 13 years, the microbial diversity decreased to the point that the community similarity had reached up to 75%–81%. This may explain the decrease of the spatial fluctuations in the communities in the disturbed soils.

The regional microbial distribution based on the abundance of soil PLFAs markers were found significantly correlated with latitude across NSW. A similar relationship at similar scale had also been found by other studies^24,35^. Along the latitude, there was also variation in agriculture activities from north to south, with more wheat in the cropping lands in the north but more ryegrass in the grazing lands in the south^34^. This latitudinal distribution of above land cover shaped the composition of the above biotic litter, regulated soil nutrients, and then influenced the balance of the microbial composition^5,7^. Correspondingly, both PC1 and PC2 were significant with latitude in the disturbed system, as strengthened the correlation between PLFAs biomarkers and the latitude (Fig. 5).

Overall, the latitudinal gradient displayed large variations of soil properties, climate and geographical patterns that all together impacted the belowground living organisms. Agricultural activities enhanced the role of latitude in shaping microbial communities. Among all factors examined in both systems, soil properties showed the most significant influences on the belowground microbial community structure. Similar results have also been reported in other areas such as Qinghai-Tibetan Plateau in China^47^ and among different landscapes across France^25^. This was also confirmed by the finding of another survey carried out in the same study area in 2013 focused on the identification of soil microorganisms based on the sequencing of soil DNA^34^. The findings by Pino demonstrated a close relationship between the soil microbial diversity patterns (with PCR-based techniques and soil DNA) and soil physicochemical properties in this region^34^. PCR-based methods offer the greatest potential for characterization of underlying populations and the understanding of changes at this high-resolution level. But PLFAs showed more power on resolving effects on the total structure of soil-living community with more statistically relevant information from the patterns analyzed^48^. In this study area, both techniques demonstrated similar conclusions about the abundance tendencies of the soil microorganisms.

## Conclusions

This study evidenced the spatial pattern of the soil microbial communities. Microbial composition showed a latitudinal gradient along the 550 mm isohyet transect. The microbial spatial distribution was influenced by soil properties, climate, topography and land use. Impacts of each factor differ amongst different microbial groups, and this biogeographic distribution was an integrated effect of all influencing factors. Among factors analyzed, soil properties, especially soil nutrients, presented the most significant contribution when structuring the spatial abundances of the soil microbial communities. Agricultural activities tend to decrease the variations of the microbial abundances but strengthen the latitude gradient of the microbial biomarkers. However, this influence was much less compared to the gradient of other factors. These results will contribute to the understanding of the soil microbial responses to environmental gradients and apply as a guidance in the use and management of land practices for future regulations.

## Methods

### Study Area

We carried out a survey in the eastern part of the state of New South Wales (NSW) in Australia. The state that exhibits a great diversity of landscape and habitats; and encompasses one of the most important agricultural areas in Australia – the *Wheat-belt East Region*. In this area (Fig. 1), we collected soil samples from different agro-ecological zones, and a variety of soils types along the 550 mm mean annual rainfall isohyet^34^. From north to south, the mean temperature in autumn 2013 – during the sampling campaign – ranged from around 27 °C to 21 °C. The distribution of soil types changed from mainly *Vertisols* in the north region to majorly *Sodosols* and some* Kandosols* towards the south, and some *Chromosols* in the very end border with the Victoria state^34^.

### Soil Samples

The soil sampling followed a 1000 km longitudinal transect (Fig. 1). This transect line was designed to extend from north to south state’s borders i.e. Queensland (latitude −28.77) to Victoria (Latitude −35.98), following the 550 mm mean annual rainfall isohyet. Along the transect, 27 locations (0 to 26) were surveyed with a separation distance of approximately 50 km each (Fig. 1) with the WGS 1984, UTM Zone 56 S geodesic system. At each location, sampling sites were determined based on their representativeness of the environmental variables (i.e., soil properties, temperature, latitude, etc.) within a circle of 20 km radius (sampling buffer). Each of those buffers was individually analyzed in terms of their environmental variability to select representative zones. These zones were defined based on a frequency distribution of key environmental variables such as climate, land cover, soil pH, salinity, soil type, among others. This information was processed in Arc/Info with data obtained from the Geophysical Archive Data Delivery System (GADDS), the Geoscience Australia (GA) and Australian Bureau Meteorology (BOM, 2009). We selected paired soil types under disturbed (i.e. cropping or grazing regions) and undisturbed conditions (i.e. natural areas, such as forest, grassland or woodlands) based on the gamma radiometric raster information. Once in the field, we confirmed that the sites had the same soil type and we sampled those sites.

Samples were collected from the top 5 cm of the soil surface within an area of 1 m × 1 m square at each ecosystem per sampling site. With three replicates at each site, 162 soil samples were collected. In the field, the samples were stored in a −4 °C refrigeration immediately after collected into the sterile falcon tubes (50 ml), and then transferred to a −21 °C freezer installation in the laboratory at the University of Sydney for further analysis. The sampling campaign was carried out from the end of March to the beginning of April in 2013, and the samples for each location were collected at once.

### Soil Analysis

#### Soil PLFAs

Phospholipid Fatty Acids (PLFAs) analysis exploits the differences in the cellular membrane composition between microbial groups to determine functionally active components of the microbial communities^41,49^. Compared with other approaches, e.g., community-level physiological profiling (CLPP) and DNA fingerprinting, PLFAs is relatively efficient and economical^50^. While PCR-based techniques or High-Throughput methods focus on communities biased by the primers selection for the amplification of a specific target on the soil DNA region, and non-living organisms (DNA from dead cells) are also included. For the high efficiency, PLFA method has been widely used for exploratory analysis of the spatial soil microbial composition^48,51^.

We used PLFAs analysis as in Buyer and Sasser^50^ using 2 g of soil. Phospholipids were isolated with solid phase extraction and derivatized into their corresponding fatty acids methyl esters (FAMEs) for gas chromatography (7890 A, Agilent Technologies, Wilmington, DE, USA) analysis. The FAMEs were identified using the MIDI PLFAD1 calibration mix and the software SHERLOCK version 6.2 (MIDI, Inc., DE, USA). For each sample, the abundance of individual fatty acid methyl esters (FAME) was expressed as nmol/g dry soil and as a percentage of total abundance. Lipid markers associated with microbial functional groups were analyzed by summing their concentrations. Groups included the gram-positive bacteria^52^, gram-negative bacteria^53,54^, unclassified bacteria^55^, actinomycetes^42,56^, saprotrophic fungi (SF)^57–59^, arbuscular mycorrhizal fungi (AMF)^53,55^, and other unclassified PLFAs^53,60^. Fungal to bacteria ratio (F:B) was calculated by dividing saprotrophic fungal PLFAs by the sum of gram-positive, gram-negative and unclassified bacterial PLFAs^55^.

#### Soil Properties and other Environment factors

Soil physicochemical characterization was carried out for each sampling site^34^. These data included a complete set of soil laboratory measurements (e.g., total N, total C, P, pH, electrical conductivity (EC), Effective Cation Exchange Capacity (ECEC), pH, clay and sand contents etc.). Environmental information was obtained in *silico* from satellite images such as topographical attributes (i.e., elevation, slope), and vegetation conditions (i.e., Normalized Difference Vegetation Index). Topographical attributes were derived from a national SRTM Digital Elevation Model, while the NDVI were derived from continental-wide Landsat images.

### Statistical Analysis

Principal Component Analyses (PCA) of the lipids was used to examine the variation in the microbial community along the latitude using all individual lipids detected and biomarker groups. PCA was conducted using JMP Pro. 11 software (SAS, 2015). The variations across the transect was analyzed by the multiple comparison across the latitude. The microbial groups were compared means across latitude for disturbed and undisturbed systems separately with the Tukey HSD analysis in JMP. Additionally, paired comparison was carried for each site to tell the difference between the ecosystems. Three replicated results from the disturbed and undisturbed soils were compared via t-test for each site.

The relationships between the biomarker groups and environmental factors were examined using Pearson Correlation Coefficient. The correlations are estimated by the Pairwise method. The level of significance of Pearson’s Correlation Coefficient (r) was determined using the Two-Tailed Test. The integrated contributions of the influencing factors were calculated using a multilinear regression. The possible influencing factors were considered altogether in the regression model. A stepwise regression approach was used to select variables that presented significant impacts to the microbial groups. The model stopping rule was minimum Bayesian Information Criterion (BIC), and the significance was judged by F ratio and p values. With the forward direction, the insignificant factors were then not considered in the next multilinear analysis. Additionally, a further correlation analysis was carried among the selected variables. The redundant ones would be removed for the next multiple linear analysis if the selected variables were highly correlated. The influencing factors of the remained effective variables would be then analyzed in the multilinear model by JMP.

## Electronic supplementary material

Supplementary Information
