# Supplementary material for: Soil Properties Drive Microbial Community Structure in a Large Scale Transect in South Eastern Australia
Source: Sci Rep. 2018 Aug 6;8:11725. doi: 10.1038/s41598-018-30005-8 (PMC6078944; doi:10.1038/s41598-018-30005-8)
Supplement: Supplementary file 1 — Supplementary Information [file 41598_2018_30005_MOESM1_ESM.pdf]

Soil Properties Drive Microbial Community Structure in a Large Scale Transect in South Eastern Australia

Pei-Pei Xue[1], Yolima Carrillo[2], Vanessa Pino[1], Budiman Minasny[1*], Alex. B. McBratney[1]

[1] School of Life and Environmental Sciences and Sydney Institute of Agriculture, The University of Sydney, Eveleigh NSW 2015, Australia

[2] Hawkesbury Institute for the Environment, Western Sydney University, Richmond NSW 2753, Australia

*** Corresponding author**. E-mail: **budiman.minasny@sydney.edu.au**

Supplementary Information

Table S1. Sample information and the microbial absolute abundances (nmol/g dry soil) of each site.

Location	Suburb	Type	Latitude	Soil Type	[Total PLFAs]	[Gram-Positive]	[Gram-Negative]	[SF]	[Actionmycetes]	[AMF]
0	*Mongindi*	Disturbed	-28.772	Vertosol	22.59	4.92	5.58	1.98	2.82	0.65
		Undisturbed	-28.772	Vertosol	37.61	9.12	9.15	2.65	5.52	0.83
1	*Garah*	Disturbed	-29.055	Vertosol	25.37	5.22	6.73	2.17	3.03	0.81
		Undisturbed	-29.055	Vertosol	37.56	7.37	9.84	3.75	4.84	1.09
2	*Moree*	Disturbed	-29.541	Vertosol	34.51	7.42	8.99	3.24	4.23	1.20
		Undisturbed	-29.541	Vertosol	42.78	9.22	10.99	4.05	4.79	1.00
3	*Rovena*	Disturbed	-29.774	Vertosol	40.94	7.68	11.33	4.50	4.61	1.57
		Undisturbed	-29.774	Vertosol	11.06	2.40	2.53	0.83	2.17	0.31
4	*Collarenebri*	Disturbed	-29.565	Vertosol	18.67	3.14	4.82	1.89	2.27	0.59
		Undisturbed	-29.566	Vertosol	41.72	7.96	11.22	4.56	4.96	1.29
5	*Rowena*	Disturbed	-29.872	Vertosol	27.97	6.10	7.37	2.73	3.68	0.82
		Undisturbed	-29.873	Vertosol	23.98	4.67	6.80	2.44	2.78	0.59
6	*Gwabegar*	Disturbed	-30.492	Sodosol	21.10	4.93	4.17	2.44	1.91	0.43
		Undisturbed	-30.493	Sodosol	22.16	4.36	5.68	2.97	2.12	0.48
7	*Urawilkie*	Disturbed	-30.753	Sodosol	21.04	5.24	4.45	1.62	2.41	0.46
		Undisturbed	-30.755	Sodosol	17.31	3.65	4.83	1.79	1.66	0.40
8	*Coonamble*	Disturbed	-31.153	Sodosol	18.65	3.82	4.35	2.17	1.91	0.43
		Undisturbed	-31.154	Sodosol	22.60	5.06	5.08	3.09	2.22	0.40
9	*Curban*	Disturbed	-31.531	Sodosol	28.69	6.14	6.51	3.55	3.00	0.82
		Undisturbed	-31.531	Sodosol	17.98	4.02	4.45	2.17	1.36	0.28
10	*Kickabil*	Disturbed	-31.939	Sodosol	24.67	4.09	6.37	3.59	1.81	0.71
		Undisturbed	-31.939	Sodosol	16.04	2.44	4.60	2.43	0.91	0.24
11	*Burroway*	Disturbed	-32.160	Ferrosol	32.96	6.57	6.97	4.81	4.09	0.84
		Undisturbed	-32.161	Ferrosol	24.93	5.16	6.39	2.87	2.85	0.63
12	*Narromine*	Disturbed	-32.453	Sodosol	27.77	5.56	6.29	3.58	2.84	0.66
		Undisturbed	-32.452	Sodosol	19.73	3.70	5.51	2.27	1.76	0.53
13	*Peak Hill*	Disturbed	-32.880	Sodosol	26.52	5.19	7.05	2.71	2.85	0.58
		Undisturbed	-32.881	Sodosol	28.72	5.77	7.66	2.97	2.66	0.70
14	*Daroobalgie*	Disturbed	-33.290	Kandosol	24.87	5.56	5.59	2.86	2.92	0.56
		Undisturbed	-33.288	Kandosol	29.98	5.62	9.16	3.35	2.91	0.80
15	*Glenelg*	Disturbed	-33.681	Calcarosol	27.02	5.90	6.90	2.62	3.14	0.69
		Undisturbed	-33.681	Ferrosol	23.11	4.77	5.79	2.21	2.43	0.37
16	*Caragabal*	Disturbed	-33.896	Ferrosol	32.14	6.45	8.11	2.76	3.04	0.66
		Undisturbed	-33.895	Dermosol	21.62	3.54	5.61	3.21	1.58	0.54
17	*Bimbi*	Disturbed	-34.014	Sodosol	29.49	5.78	7.20	3.29	3.01	0.53
		Undisturbed	-34.013	Sodosol	29.96	6.35	7.21	3.08	3.00	0.44
18	*Stockbingal*	Disturbed	-34.437	Sodosol	32.25	8.15	6.77	2.72	3.61	0.37
		Undisturbed	-34.439	Sodosol	23.52	5.05	6.03	2.50	2.11	0.26
19	*Combaning*	Disturbed	-34.572	Ferrosol	31.10	6.91	7.14	3.21	3.22	0.40
		Undisturbed	-34.570	Calcarosol	34.88	5.58	7.70	4.55	2.37	0.11
20	*Wantiool*	Disturbed	-34.862	Kandosol	35.74	7.10	8.64	3.71	3.86	0.93
		Undisturbed	-34.862	Kandosol	24.63	5.64	5.16	2.32	2.67	0.31
21	*Yathella*	Disturbed	-34.960	Ferrosol	28.17	5.84	7.31	2.93	2.55	0.51
		Undisturbed	-34.958	Dermosol	27.02	5.46	7.21	2.50	2.84	0.60
22	*Forest Hill*	Disturbed	-35.181	Dermosol	22.75	4.58	5.72	2.07	2.54	0.77
		Undisturbed	-35.181	Dermosol	26.39	6.01	6.06	2.68	2.58	0.40
23	*The Rock*	Disturbed	-35.296	Sodosol	30.26	7.11	6.66	2.68	3.92	0.44
		Undisturbed	-35.296	Sodosol	26.64	5.42	7.16	3.12	2.38	0.45
24	*Munyabla*	Disturbed	-35.485	Sodosol	31.79	6.81	7.26	4.09	2.31	0.52
		Undisturbed	-35.485	Sodosol	27.71	6.10	6.40	2.60	2.89	0.53
25	*Brocklesby*	Disturbed	-35.797	Sodosol	42.12	8.78	10.81	4.54	3.77	1.32
		Undisturbed	-35.799	Sodosol	38.72	7.26	10.50	4.00	3.11	0.77
26	*Howlong*	Disturbed	-35.976	Chromosol	44.88	8.72	12.32	5.53	4.34	1.28
		Undisturbed	-35.975	Chromosol	49.85	9.57	14.78	4.39	4.49	1.30

* The absolute abundances of the microbial groups were the means of three replicates quantified by their PLFAs biomarkers. PLFAs= Phospholipid Fatty Acids; SF= saprotrophic fungi; AMF=arbuscular mycorrhizal fungi.

Table S2: Multiple comparison of the microbial mean absolute abundances (nmol/g dry soil) through Tukey HSD analysis within all the sites along the gradient in the disturbed and undisturbed ecosystems.

Location	Total PLFAs (nmol/g dry soil)		Gram Negative (nmol/g dry soil)		Gram Positive (nmol/g dry soil)		Actinomycetes (nmol/g dry soil)		SF (nmol/g dry soil)		AMF (nmol/g dry soil)	
	Undisturbed	Disturbed	Undisturbed	Disturbed	Undisturbed	Disturbed	Undisturbed	Disturbed	Undisturbed	Disturbed	Undisturbed	Disturbed
0	A B C D	D E F	B C D E	D E F G	A B C	C D E F G	A	E F G H I	A B C D E	F G H	B C D	E F G
1	A B C D	D E F	B C D	D E F G	A B C D E	B C D E F G	A B	C D E F G H I	A B C D	F G H	A B	D E F G
2	A B	A B C D	A B C	A B C D	A B	A B C D	A B	A B C	A B C	B C D E F G H	A B C	A B C D
3	H	A B C	G	A B	G	A B C	D E	A	E	A B C D	F G H	A
4	A B C	F	A B	E F G	A B C D	G	A B	H I	A	G H	A	E F G
5	D E F G H	C D E F	B C D E F G	C D E F G	D E F G	A B C D E F G	C D	A B C D E F	B C D E	E F G H	C D E F G	D E F G
6	E F G H	E F	D E F G	G	D E F G	C D E F G	D E	I	A B C D	E F G H	D E F G H	E F G
7	G H	E F	E F G	F G	F G	B C D E F G	D E	G H I	D E	H	E F G H	E F G
8	D E F G H	F	E F G	G	D E F G	F G	D E	I	A B C D	F G H	E F G H	E F G
9	G H	C D E F	F G	D E F G	E F G	A B C D E F G	D E	C D E F G H I	C D E	B C D E F G	G H	D E F G
10	G H	D E F	E F G	D E F G	G	E F G	E	I	B C D E	B C D E F	G H	E F G
11	D E F G H	A B C D E	C D E F G	D E F G	D E F G	A B C D E F	C D	A B C D	A B C D	A B	C D E F G	C D E F
12	G H	D E F	D E F G	D E F G	F G	B C D E F G	D E	D E F G H I	C D E	B C D E F	D E F G	E F G
13	B C D E F G	D E F	B C D E F	D E F G	B C D E F G	B C D E F G	D E	D E F G H I	A B C D	E F G H	B C D E F	E F G
14	B C D E F G	D E F	B C D E	D E F G	B C D E F G	B C D E F G	C D	D E F G H I	A B C D	D E F G H	B C D E	E F G
15	D E F G H	C D E F	D E F G	C D E F G	D E F G	A B C D E F G	D E	B C D E F G H I	C D E	E F G H	F G H	D E F G
16	F G H	A B C D E	D E F G	B C D E F	F G	A B C D E F	D E	C D E F G H I	A B C D	E F G H	D E F G	E F G
17	B C D E F G	B C D E F	B C D E F	C D E F G	A B C D E F	A B C D E F G	C D	C D E F G H I	A B C D	B C D E F G	D E F G H	E F G
18	D E F G H	A B C D E	D E F G	D E F G	D E F G	A B	D E	A B C D E F G	B C D E	E F G H	G H	G
19	A B C D E F	B C D E F	B C D E F	C D E F G	C D E F G	A B C D E	D E	B C D E F G H	A	B C D E F G H	H	F G
20	D E F G H	A B C D E	E F G	A B C D E	B C D E F G	A B C D E F	D	A B C D E F	C D E	A B C D E F G	F G H	B C D E
21	C D E F G	C D E F	B C D E F	C D E F G	D E F G	A B C D E F G	C D	F G H I	B C D E	C D E F G H	C D E F G	E F G
22	D E F G	D E F	D E F G	D E F G	A B C D E F G	D E F G	D E	F G H I	A B C D E	F G H	E F G H	D E F G
23	C D E F G	B C D E F	B C D E F G	D E F G	D E F G	A B C D E	D E	A B C D E	A B C D	E F G H	D E F G H	E F G
24	B C D E F G	A B C D E F	C D E F G	C D E F G	A B C D E F	A B C D E F	C D	H I	A B C D E	A B C D E	D E F G H	E F G
25	A B C D E	A B	A B C D	A B C	A B C D E F	A	B C D	A B C D E F	A B C D	A B C	B C D E F	A B
26	A	A	A	A	A	A	A B C	A B	A B	A	A	A B C

* PLFAs= Phospholipid Fatty Acids; SF= saprotrophic fungi; AMF=arbuscular mycorrhizal fungi, F: B was calculated by dividing saprotrophic fungal PLFAs by the sum of gram-positive, gram-negative and unclassified bacterial PLFAs.
* The letters indicate the differences of the mean absolute abundances of each site, and two sites share the different letters denote the significant difference between each other.

Table S3. Linear correlation coefficients between the microbial absolute abundances (nmol/g dry soil) of each group and the environmental factors.

Undisturbed Ecosystem															
	Total C	Total N	C:N	P (Colwell)	K (Colwell)	EC	ECEC	pH ($CaCl_2$)	Clay Content	Sand	Latitude	Elevation	Surface Temp	slope	NDVI
Total PLFAs	0.36	0.42	-0.15	***0.50***	0.37	0.11	***0.50***	0.23	0.32	-0.37	-0.09	-0.34	0.12	-0.19	-0.11
Gram-Positive	0.20	0.29	-0.33	0.47	0.38	0.14	***0.49***	0.29	0.39	-0.37	-0.02	-0.38	0.20	-0.23	-0.21
Gram-Negative	0.33	0.39	-0.12	***0.49***	0.31	0.07	0.48	0.26	0.32	-0.37	-0.08	-0.41	0.10	-0.21	-0.10
Actinomycetes	0.01	0.09	-0.38	***0.61***	0.47	0.13	***0.61***	***0.55***	***0.63***	-0.43	0.28	***-0.57***	***0.49***	-0.38	-0.40
SF	***0.49***	0.47	0.20	0.36	0.26	-0.08	0.32	-0.03	0.09	-0.19	-0.14	-0.05	-0.06	0.10	0.07
AMF	-0.03	0.05	-0.26	***0.51***	0.28	-0.02	0.47	***0.50***	0.44	-0.25	0.24	***-0.59***	0.39	-0.32	-0.27
F:B	0.14	0.00	***0.72***	-0.37	-0.35	-0.40	-0.43	***-0.57***	-0.42	0.42	-0.09	***0.53***	-0.28	***0.54***	0.28

Disturbed Ecosystem															
	Total C	Total N	C:N	P (Colwell)	K (Colwell)	EC	ECEC	pH ($CaCl_2$)	Clay Content	Sand	Latitude	Elevation	Surface Temp	slope	NDVI
Total PLFAs	***0.58***	***0.60***	-0.04	0.26	0.22	0.14	0.13	-0.03	-0.03	0.01	-0.45	-0.04	-0.18	0.14	0.02
Gram-Positive	***0.55***	***0.59***	-0.13	0.06	0.23	0.04	0.06	-0.23	-0.04	-0.09	***-0.48***	0.02	-0.31	0.19	0.09
Gram-Negative	***0.57***	***0.57***	-0.04	0.36	0.28	0.31	0.31	0.19	0.09	-0.06	-0.34	-0.18	-0.03	0.06	-0.12
Actinomycetes	0.35	0.38	-0.20	0.00	0.43	0.17	0.37	0.13	0.33	-0.28	-0.14	-0.14	0.02	0.09	-0.12
SF	0.48	0.46	0.08	0.39	-0.02	0.02	-0.06	-0.05	-0.18	0.25	-0.40	-0.07	-0.11	-0.02	0.22
AMF	0.18	0.17	-0.06	0.34	0.25	0.25	***0.52***	***0.52***	0.33	-0.11	0.09	-0.45	0.40	-0.11	-0.22
F:B	-0.01	-0.06	0.25	0.29	-0.35	-0.23	-0.36	-0.09	-0.32	0.53	-0.09	0.05	0.04	-0.14	0.39

* PLFAs= Phospholipid Fatty Acids; SF= saprotrophic fungi; AMF=arbuscular mycorrhizal fungi. EC= Electrical Conductivity; ECEC= Cation Exchange Capacity; NDVI= Normalized Difference Vegetation Index.

* Significant correlations are highlighted in ***Italic bold*** ($P \leq 0.01$) in accordance with a Pearson's paired sample association test.

Table S4. Linear correlation coefficients between the microbial relative abundances (%) of each group and the environmental factors.

Undisturbed Ecosystem															
	Total C	Total N	C:N	P (Colwell)	K (Colwell)	EC	ECEC	pH ($CaCl_2$)	Clay Content	Sand	Latitude	Elevation	Surface Temp	slope	NDVI
Gram-Positive	-0.39	-0.27	***-0.71***	0.01	0.14	0.19	0.04	0.28	0.17	-0.07	0.16	-0.14	0.20	-0.16	-0.26
Gram-Negative	0.00	0.02	0.24	0.08	-0.16	-0.27	0.04	0.03	0.09	-0.14	0.06	-0.34	-0.02	-0.10	0.06
Actinomycetes	-0.37	-0.30	***-0.58***	0.40	0.39	0.31	0.48	***0.77***	***0.63***	-0.29	***0.52***	***-0.55***	***0.64***	-0.40	***-0.53***
SF	0.11	-0.02	***0.72***	-0.37	-0.37	-0.47	-0.45	***-0.56***	-0.44	0.40	-0.06	***0.50***	-0.30	***0.53***	0.28
AMF	-0.37	-0.30	-0.25	0.31	0.10	-0.11	0.28	***0.58***	0.38	-0.06	0.47	***-0.58***	***0.52***	-0.31	-0.29
PC 1	0.36	0.27	0.43	***-0.61***	-0.39	-0.08	***-0.57***	***-0.84***	***-0.72***	0.31	***-0.69***	***0.73***	***-0.74***	0.43	***0.56***
PC 2	0.09	-0.04	***0.76***	-0.16	-0.44	***-0.51***	-0.23	-0.36	-0.13	0.11	0.08	0.06	-0.02	0.14	0.11

Disturbed Ecosystem															
	Total C	Total N	C:N	P (Colwell)	K (Colwell)	EC	ECEC	pH ($CaCl_2$)	Clay Content	Sand	Latitude	Elevation	Surface Temp	slope	NDVI
Gram-Positive	-0.01	0.04	-0.21	***-0.53***	0.04	-0.30	-0.19	***-0.59***	-0.04	-0.27	-0.09	0.14	-0.34	0.07	0.13
Gram-Negative	0.20	0.18	0.02	0.44	0.32	***0.58***	***0.60***	0.71	0.32	-0.14	0.09	-0.36	0.38	-0.12	-0.40
Actinomycetes	-0.26	-0.24	-0.30	-0.37	0.39	0.11	0.48	0.34	***0.63***	***-0.52***	0.44	-0.24	0.36	-0.08	-0.27
SF	0.03	-0.01	0.21	0.32	-0.32	-0.19	-0.31	-0.07	-0.30	***0.51***	-0.11	0.00	0.05	-0.19	0.38
AMF	-0.23	-0.25	-0.05	0.31	0.16	0.27	***0.64***	***0.81***	***0.49***	-0.14	***0.49***	***-0.60***	***0.71***	-0.26	-0.31
PC 1	0.46	0.48	0.10	-0.04	-0.24	-0.17	***-0.77***	***-0.82***	***-0.72***	0.29	***-0.76***	***0.68***	***-0.87***	0.45	0.47
PC 2	-0.15	-0.21	0.17	0.30	0.13	0.28	***0.53***	***0.75***	0.42	-0.16	***0.51***	***-0.50***	***0.67***	-0.29	-0.26

* SF = saprotrophic fungi; AMF = arbuscular mycorrhizal fungi. EC = Electrical Conductivity; ECEC = Cation Exchange Capacity; NDVI = Normalized Difference Vegetation Index. PC1 and PC2 was the values of first and second principal components from Principal Component Analysis of molecular weight percentages of whole communities' PLFA profiles along the transect
* Significant correlations are highlighted in ***Italic bold*** (P≤0.01) in accordance with a Pearson's paired sample association test.
